# Cell Phenotype Transitions in Cardiovascular Calcification

**DOI:** 10.3389/fcvm.2018.00027

**Published:** 2018-03-26

**Authors:** Luis Hortells, Swastika Sur, Cynthia St. Hilaire

**Affiliations:** Division of Cardiology, Department of Medicine, and the Pittsburgh Heart, Lung, and Blood Vascular Medicine Institute, University of Pittsburgh, Pittsburgh, PA, United States

**Keywords:** vascular calcification, valvular calcification, cell phenotype transition, vascular smooth muscle cell, endothelial cell, valve interstitial cell

## Abstract

Cardiovascular calcification was originally considered a passive, degenerative process, however with the advance of cellular and molecular biology techniques it is now appreciated that ectopic calcification is an active biological process. Vascular calcification is the most common form of ectopic calcification, and aging as well as specific disease states such as atherosclerosis, diabetes, and genetic mutations, exhibit this pathology. In the vessels and valves, endothelial cells, smooth muscle cells, and fibroblast-like cells contribute to the formation of extracellular calcified nodules. Research suggests that these vascular cells undergo a phenotypic switch whereby they acquire osteoblast-like characteristics, however the mechanisms driving the early aspects of these cell transitions are not fully understood. Osteoblasts are true bone-forming cells and differentiate from their pluripotent precursor, the mesenchymal stem cell (MSC); vascular cells that acquire the ability to calcify share aspects of the transcriptional programs exhibited by MSCs differentiating into osteoblasts. What is unknown is whether a fully-differentiated vascular cell directly acquires the ability to calcify by the upregulation of osteogenic genes or, whether these vascular cells first de-differentiate into an MSC-like state before obtaining a “second hit” that induces them to re-differentiate down an osteogenic lineage. Addressing these questions will enable progress in preventative and regenerative medicine strategies to combat vascular calcification pathologies. In this review, we will summarize what is known about the phenotypic switching of vascular endothelial, smooth muscle, and valvular cells.

## Introduction

In bone formation, there are two different ossification processes, intramembranous ossification and endochondral ossification (1). During intramembranous ossification, the mineral hydroxyapatite is produced by osteoblasts and secreted into the dense network of extracellular matrix (ECM) proteins, together which harden to form a mineralized bone structure. Endochondral ossification involves hyaline cartilage and chondrocytes as a precursor for the hydroxyapatite nesting. Calcification in areas other than bone or tooth formation is pathologic, developing in the ECM of soft tissues, where osteoblasts do not reside. Ectopic calcification was once considered a passive and degenerative process, but it is now recognized as an active biological process which shares many features of physiological bone formation and remodeling, however the precise mechanisms inducing and propagating pathological calcification are not completely understood. The resulting pathology from ectopic calcification can induce or exacerbate a variety of disease states.

Calcification of the cardiovascular system is one of the most frequent expressions of ectopic calcification, and the sites exhibiting calcification include the myocardium, heart valves, and the large and small arteries of the body (2–4). Myocardial calcification presents in two main forms: metastatic and dystrophic. The former is associated with aberrations in calcium homeostasis and is commonly found in patients with chronic kidney disease or kidney failure, as well as hyperparathyroidism (5, 6). Dystrophic myocardial calcification is more prevalent than metastatic, and occurs as a result of injury due to events such as myocardial infarction or infection (7, 8). Calcification of the aortic valve, termed calcific aortic valve disease (CAVD), encompasses a wide spectrum of pathology, from the stiffening of the leaflets (aortic sclerosis) to the presence of calcification that impairs leaflet movement and reduces blood flow (aortic stenosis). CAVD represents an ever-growing health burden associated with substantial costs (9, 10). Aortic valves are composed of three leaflets made up of three layers: the collagen-rich fibrosa lines the aortic side, the proteoglycan and glycosaminoglycan-rich spongiosa in the middle layer, and the elastin and collagen-rich ventricularis on the side of the left ventricle (3). Valve endothelial cells (VECs) cover the surface of the leaflets while valve interstitial cells (VICs) reside in all three layers. While both VECs and VICs can calcify, nodules of calcification originate in the fibrosa along the aortic side (11). In the arteries, calcification is divided in two main forms: intimal and medial. While the advanced stages of calcification in either arterial layer can invade into the other, the origin and course of these pathologies is distinct. Calcification of the intima is derived from atheroma plaque formation and is driven in part by necrosis, inflammation, and changes in endothelial cells (12), while early stages of medial calcification are not driven by inflammation but rather a breakdown of extracellular matrix, vascular smooth muscle cell (VSMC) phenotypic change (13), as well as an accumulation of extracellular matrix vesicles that are loaded with a variety of proteins, microRNAs, and the calcium and phosphate building blocks necessary for mineralization (14). In the field of vascular calcification, atherosclerotic intimal calcification is more widely recognized and better studied, while non-atherosclerotic medial calcification, which commonly occurs in patients with diabetes, renal disease, or hypertension, and several genetic diseases, has been less studied and therefore the processes that drive  this pathology are less understood (4).

In this review, we will focus on the contribution of cellular fate, and how fully differentiated cells can revert to an immature state and then acquire an osteoblastic phenotype that drives calcification pathogenesis in cardiac tissues, aortic valves, and medial-layer calcification. Similarto osteogenic transitions, chondrocytic phenotype changes have also been identified during cardiovascular ossification pathobiology (15, 16). Recently, various studies have focused on the role of cell phenotype switching. In addition to changes in cell function, this phenomenon implies global transcriptional modifications that lead to the aberrant activation of genes involved in the calcification process.

## Fibroblast to Myofibroblast to Osteoblast-Like Cell

Studies in murine models have identified that cardiac fibroblasts make up close to 25% of the heart tissue (17). While not possessing electrical or contractile functions themselves, cardiac fibroblasts can couple to cardiomyocytes to aid in the propagation of electrical signals, maintain extracellular matrix homeostasis, and secrete cytokines and chemokines to modulate the immune system (18, 19). After injury, these fibroblasts exhibit functions to remodel the ECM, alter chemical and mechanical signals, participate in angiogenesis, and contribute to fibrosis (20, 21). Cardiac fibroblasts, like fibroblasts of other tissues, can acquire a “myofibroblast” phenotype, a state which shares some of the features seen in smooth muscle cells, including the ability to contract, the acquisition of smooth muscle cell markers such as α-smooth muscle actin (SMA-α), and secretion of ECM components (21, 22). It is well known that ectopic calcification in soft-tissue occurs at sites of injury, near the resulting scar tissue generated from fibrotic remodeling (23). Considering this, elegant experiments by Pillai et al. sought to determine whether cardiac fibroblasts are the source of cardiac calcifications (24). *In vitro* studies showed that with treatment of medium that differentiates mesenchymal stem cells into osteoblasts (often referred to as osteogenic media) both murine and human cardiac fibroblasts, but not endothelial cells, could be induced to calcify. *In vivo* lineage tracing experiments in a murine line prone to develop myocardial calcification show that cardiac fibroblasts reside amongst the hydroxyapatite minerals in fibrotic areas, and further analysis identified osteogenic signatures, such as the master osteogenic transcription factor *Runx2* (24). This work also highlights the important and complex role of inorganic phosphate (Pi) and pyrophosphate (PPi) homeostasis. Pi is a building block of mineralization, while PPi is generally considered an endogenous calcification inhibitor. Enzymes regulating this homeostasis include tissue non-specific alkaline phosphatase (TNAP), which metabolizes PPi into Pi, and ectonucleotide pyrophosphatase/phosphodiesterase-1 (ENPP1) which breaks down ATP into AMP and PPi (25). The disease Generalized Arterial Calcification of Infancy (GACI) is caused by homozygous inactivating mutations in this gene (26, 27). However, Pillai et al noticed that injured hearts presenting with calcification also showed increased expression of ENPP1. While hydroxyapatite is the most common chemical formulation found in ectopic calcification, other chemical formulations exist (4), including calcium pyrophosphate dihydrate (CPPD) (28). Indeed, the authors found CPPD minerals in calcified cardiac tissue (24), suggesting that perhaps ENPP1 was driving pathogenesis. A small molecule ENPP1 inhibitor was used and prevented this cardiac calcification (24). These results highlight the complicated dynamics of Pi/PPi homeostasis and the importance of knowing the chemical content of ectopic calcification when considering therapeutics. The study also clearly illustrates the ability of a fibroblast cell to acquire an osteogenic phenotype, but further work is needed to detail the step-wise progression that triggers differentiation from a myofibroblast-state down an osteogenic lineage.

The aortic valve also contains a fibroblast-like cell, called the valve interstitial cell (VIC). VICs populate all three layers of the valve and reside in a quiescent state. The aortic valve is a dynamic structure that controls the unidirectional flow of blood from the left ventricle to the aorta. In systole, valves open against the wall of the aorta, and the reverse pressure gradient in diastole induces them to unfurl and stretch out toward the center of the aortic annulus, forming a seal to prevent regurgitation. Every heartbeat induces this movement which exposes the valve cells and their surrounding extracellular matrix to an array of stresses (e.g., mechanical, shear, inflammatory). Mechanical and inflammatory stresses alone can induce a transcriptionally permissive chromatin structure (29, 30). These stresses are also thought to contribute to the early events that drive VICs to transition from a quiescent state to the activated myofibroblast state, which can go on to become calcifying osteoblast-like VICs (3, 11, 31–34).

It is well-established that osteogenic genes such as *Runx2, osteocalcin, and TNAP* are all upregulated in calcifying cells (32, 35, 36). The induction of these osteogenic genes in myofibroblasts is reminiscent of the differentiation of a mesenchymal stem cell (MSC) into an osteoblast (37–39). When MSCs themselves are seeded onto valve scaffolds and cultured under pulsatile flow conditions they acquire a myofibroblast-like phenotype, suggesting that exposure to mechanical and flow forces can drive progenitor cells to differentiate down the osteogenic lineage (40). In line with myofibroblast plasticity, VICs can exhibit the MSC/pericyte-like function of providing structural support to valve endothelial networks (41). *In vitro* co-culture assays in matrigel found that VICs possess chemo-attractive properties and wrap around sprouts of valve endothelial cells (VECs). Together these observations suggest that activated myofibroblasts can behave and respond to stimuli like MSC-like cells that are then further induced to upregulate expression of osteogenic genes (32, 34, 37, 42).

*In vitro* VICs acquire an activated myofibroblast-like state in part via increasing expression of TGF-β, which drives their proliferation, migration, and expression of the myofibroblast marker SMA-α (43). Activated VICs themselves alter the mechanical properties of the valve, creating a stiffer environment (44, 45). Elastic properties of the ECM also influence valve cell biology as stiffness promotes a calcific phenotype (33). Culturing VICs on a stiffer matrix promotes osteogenic differentiation, and specific substrates such as fibrin, heparin, and laminin induce the osteogenic transition of VICs into calcifying cells. In osteoblasts, ENPP1 generates PPi, which when hydrolyzed generates Pi with subsequent formation of hydroxyapatite (46), yet interestingly and similar to what was found in cardiac calcification, ENPP1 has also been found to be highly expressed in calcific aortic valve disease and in VICs (47). This release ATP promotes VICs survival, but in disease tissues upregulation of ENPP1 depletes the extracellular pool of ATP and thus promotes mineralization in VICs by promoting apoptosis (47, 48).

Inflammation also contributes to calcification pathogenesis, and inflammatory cells are found within and surrounding the calcified areas in the valve and heart (7, 49). Murine studies show that recruitment of immune cells is an early event in CAVD pathogenesis (50). And like the effects of mechanical stretch, inflammatory cells, such as mast cells, can also contribute to remodeling the ECM via the release of proteases and growth factors known to drive both physiological and pathophysiological calcification (15). The contribution of inflammation to the early progression of osteogenesis on vascular cells was illustrated *in vivo* in the valves and arteries in the atherosclerotic *ApoE* knockout model (51). This study followed the temporal association of inflammation and calcification in atherosclerosis and found that inflamed areas exhibited high levels of the key mineralization enzyme, alkaline phosphatase, before microscopic evidence of calcification. PET imaging techniques found a similar temporal association in calcified foci in human thoracic aortas (52). The inflammatory cytokines TNF-α induced early differentiation of human bone marrow-derived MSCs into calcifying osteoblast-like cells, illustrating that inflammatory pathway activation can prime a cell to become osteogenic (53). Additionally, TNF-α signals stimulated by high fat diet-induced obesity and type II diabetes mellitus promotes aortic *Msx2* expression, a transcription factor in the BMP signaling pathway, and enhances pro-calcific arterial *Msx2-Wnt* cascades (54). Together this data suggests that in atherosclerotic calcification, inflammation precedes calcification; subsequent studies should delineate the role of inflammation and inflammatory signaling pathways in driving pro-osteogenic transcriptional and epigenetic changes.

## Endothelial to Mesenchymal Transition

Cells of various developmental origins come together to create the tissues that comprise the adult vasculature. Angioblasts are the developmental precursors to endothelial cells. Once endothelial cells are specified *de novo* vasculogenesis can occur, though the precise molecular cues regulating these early processes *in vivo* have not yetbeen fully characterized (55). During development, some structures are derived from the de-differentiation of endothelial cells, a process referred to as endothelial-to-mesenchymal transition (EndMT). For example the endocardial cushion tissue, which is the precursor of the semilunar valves of the heart, are derived from cells that undergo EndMT (56). Endothelial cells form a barrier along the lumen of vessels that is held intact by endothelial-specific proteins which form tight junctions and connections between adjacent endothelial cells (57). In EndMT, expression of these markers diminishes and endothelial cells lose cell-to-cell connections, enabling their migration and proliferation, as well as trans-differentiation. The TGF-β superfamily of cytokines, which includes both TGF-βs and BMPs, has several important and broad roles such as regulating cell growth and multiplication, differentiation or apoptosis, and EndMT. Embryonic EndMT processes are regulated by TGF-β signaling (58) via the upregulation of transcription factors such as Snail, which drives the detachment of the endothelial cells, promoting their phenotype switch (59).

While EndMT is a developmental program, it is also activated after vascular injury and stress, such as vein-graft remodeling and neointima formation (60), or in disease states such as atherosclerotic plaque development and progression (61), cardiac fibrosis (62) and CAVD pathogenesis (63). In developmental and pathogenic EndMT, endothelial cells not only lose their markers, but gain expression of mesenchymal progenitor cell genes such as *Snail1, Twist1, Msx1/2, and Sox9,* indicative of phenotypic transition (64), suggesting that they are switching from their fully differentiated phenotype into a pluripotent-like state that has the ability to then de-differentiate down another mesenchymal-derived lineage.

The clearest evidence that EndMT contributes to vascular calcification is found in the disease fibrodysplasia ossificans progressive (FOP), where patients develop calcification in the microvasculature in the soft tissue. FOP stems from mutations that cause constitutive activation of the TGF-β superfamily receptor *ALK2 *(65), which propagates BMP4 signaling. Calcified lesions in FOP patients exhibit evidence of EndMT, as both endothelial (vWF, VE-Cadherin), mesenchymal (Sox9), and osteoblast (osteocalcin) proteins are co-expressed on cells. This pattern mimics a mouse model of the disease. *In vitro* experiments using endothelial cells treated with TGF-β or BMP4 showed these signals induce expression of mesenchymal cell markers and allow these cells to behave like true MSCs, differentiating down the adipogenic, chondrogenic, and osteogenic lineages (66). Thus, in FOP patients the constitutively active ALK2 primes vascular endothelial cells to transition and acquire mesenchymal-like properties, enabling them to differentiate into osteoblast-like calcifying cells. This would suggest a step-wise progression from fully-differentiated cell to a cell with a progenitor-like state that is then directed down an osteogenic lineage.

Aberrant BMP signaling and EndMT also contribute to medial calcification in larger conduit vessels. Keutel syndrome, a rare autosomal recessive disease, stems from mutations in the gene *Matrix GLA Protein (MGP)*, and these patients develop ectopic calcification in soft tissue throughout the body, including the vasculature (67). MGP acts as a potent inhibitor of vascular calcification via binding to and quenching BMP signaling (68, 69). The importance of MGP’s inhibitory activities is clearly apparent in the *MGP-*knockout mouse model, which develops extensive and severe medial-layer calcification in the large arteries and results in death a few months after birth (70). The endothelium of *MPG*-knockout mice exhibits endothelial specific markers (CD31, vWF) as well as markers of multipotency (Sox2, Nanog, Oct4) and osteogenesis (Osterix). Specifically, this study found that expression of multipotent markers occurred before expression of osteogenic genes (71). Key to these trans-differentiation events is the Yamanaka factor Sox9, as endothelial-specific deletion of this gene inhibits calcification on both the *MGP* and diabetic *Ins2^Akita/+^* backgrounds (72). This further suggests that in the transition of cells from their fully-differentiated state to an osteogenic state, cells pass through a multipotent stem cell-like state.

EndMT also contributes to CAVD pathobiology (73). During embryogenesis, valve endothelial cells (VECs) sit atop a layer of matrix referred to as the cardiac jelly. A subset of VECs are stimulated to undergo EndMT and migrate into this jelly which forms the cardiac cushions. By processes that are still not thoroughly understood, cardiac cushions morph into the leaflets, and the cells within these new structures differentiate into VICs (73). As mentioned above, the layers of the valve are rich with collagens, elastin, proteoglycans, and glycosaminoglycans, and it is well-established that these ECM proteins can initiate and propagate signaling events. For example, the glycosaminoglycans chondroitin sulfate and hyaluronic acid can drive EndMT in healthy adult VECs in a 3D *in vitro* culture system (74). The constant movement of the valves exposes the leaflets to both mechanical and shear forces. VECs are directly exposed to these stresses, which are sufficient to induce a healthy VEC to undergo EndMT in 3D *in vitro* models (75). The severity of these mechanical forces can elicit differential effects; low levels of strain induced Wnt signaling in a 2D model using sheep VECs, while high levels of cyclic strain induced TGF-β signaling (76). While TGF-β is known to drive EndMT in the development of the valves, a study looking for the early drivers of EndMT identified that inflammatory cytokines induce EndMT via Akt/NF-κB activation in both embryonic and adult VECs, but that TGF-β signaling only induced EndMT in the embryonic cells (77). Mechanical stress signals may trigger the initiation of EndMT, and with the acquisition of an MSC-like state a cell may be more readily primed to transdifferentiate into a calcifying cell.

## Synthetic Smooth Muscle Cells to Osteoblast-Like Cells

Vascular smooth muscle cells (VSMCs) comprise the medial-layer of blood vessels. They are organized in concentric circular layers along the elastic lamina, and are surrounded by the extracellular matrix and contractile fibers. In healthy adult tissues VSMCs reside in a quiescent, contractile state, commonly referred as a contractile phenotype (78), but in diseased or damaged arterial beds, VSMCs can switch from this fully-differentiated state to a proliferative one, referred to as the synthetic phenotype. Synthetic VMSCs have diminished expression of contractile proteins such as smooth muscle α-actin (*ACTA2*) or smooth muscle myosin heavy chain (*Myh11*) (79, 80); this dedifferentiation also occurs in the development of ectopic vascular calcification (81). With higher proliferative capacity and protein synthesis, as well as a progressive loss of contractile proteins, synthetic VSMCs seem to have features resembling myofibroblasts and MSCs (82). Indeed, an *in vitro* study that compared the gene expression profile of calcifying VSMCs and MSCs differentiating into osteoblasts found that while the overall transcriptional program differed between these groups, a sub-set of genes that make ECM proteins and catalyze biomineralization were shared between the two cell types (83). While this demonstrates that VSMCs undergo a transcriptional shift, this study was performed at the time point when both VSMCs and MSCs produced calcified matrix (after 25 days of osteogenic stimulation), and it remains unclear if in this process VSMCs undergo a stepwise process where they acquire a pluripotent MSC-like state before further differentiating into a calcifying cell.

VSMCs in the various vascular beds are derived from different embryonic origins. Fate mapping was first used to identify that the abdominal aorta SMCs come from splanchnic mesoderm, thoracic aorta SMCs from somatic mesoderm, aortic arch SMCs from neural crest, and coronary artery SMCs from the proepicardium (84). More recently, elegant fate-mapping was performed in murine models and found that VSMCs derived from the cardiac neural crest extended from the aortic root through the aortic arch, while VSMCs derived from the second heart field localized to the ascending aorta (85). More importantly, this study identified heterogeneity in the developmental origin of VSMCs in the ascending aorta; VSMCs of the inner laminar regions close to the intima are derived from cardiac neural crest, while cells along the outer laminar area along the adventitial side of the vessel wall are derived from the second heart field (85). The different developmental origins of VSMCs may be an important key to understanding cardiovascular disease pathogenesis. In the case of vascular calcification, VSMCs in atherosclerotic lesions from the coronaries have a higher propensity to calcify than VSMCs of the aortic wall (86). Indeed, our independent studies using primary human VSMCs isolated from the coronary and aorta of the same patient show that coronary VSMCs readily calcify but aortic VSMCs do not (unpublished). Additionally, under similar calcifying conditions, Leroux-Berger et al. showed that the VSMCs in the aortic arch region, which are neural crest derivatives, calcify earlier than the VSMCs in the regions flanking both sides of the aortic arch, which are of mesodermal origin. This supports the idea that the embryonic origin influences the ability of a cells to calcify  (87). Further highlighting the distinctions in the calcification potential of VMSCs residing in different vascular beds is the genetic disease Arterial Calcification due to Deficiency of CD73 (ACDC; also, called CALJA) (88). Patients with ACDC develop medial-layer vascular calcification that is localized to their lower-extremity arteries and is dependent on the upregulation of the mineralizing enzyme, TNAP (89). Pathological samples showed that calcification appears to initiate along the internal elastic lamina, which is fragmented and duplicated (88, 90). This data is highly suggestive that the ability of healthy VSMCs to transdifferentiate into calcifying cells is influenced by the developmental origin. Further exploration of this hypothesis could uncover novel epigenetic signatures that prime cells to transdifferentiate into osteoblast-like cells capable of producing calcified matrix.

Factors such as high concentrations of extracellular phosphate and calcium, oxidized lipoproteins and reactive oxygen species, and inflammatory cytokines help drive VSMCs toward a calcifying phenotype in both atherosclerotic and non-atherosclerotic calcification (91–96). The role of TGF-β superfamily signaling, which includes TGF-β as well as bone morphogenic protein (BMP) cytokines, is well characterized in the regulation of skeletal development and bone homeostasis (97), and not surprisingly, these pathways are also upregulated in the calcification of VSMCs. Advanced atherosclerotic lesions exhibit increased levels of TGF-β and bone-like structures (98, 99), and can induce osteogenic differentiation and calcification of VSMCs *in vitro* (100, 101). As in endothelial cells mentioned above, in VSMCs, TGF-β signaling is kept in check by MGP (70). MGP exerts its anti-calcific effects via repressing TGF-β signaling and allowing Wnt/Notch signaling to keep VSMCs in their fully-contractile state (16). Another TGF-β family member, BMP2, stimulates VMSCs to uptake inorganic phosphate and induces transcription of the osteogenic transcription factor *RUNX2 *(102). In addition to activation of osteogenic transcriptional programs, TGF-β signaling contributes to the secretion of calcifying extracellular vesicles that accumulate in the extracellular matrix of VSMCs (103). While TGF-β family cytokines, as well as other stimuli, induce VSMCs to calcify, it is still not clear if the phenotypic switch to a calcifying cell happens directly from the mature, fully differentiated state, or if there is an intermediate MSC-like cell that requires the proper signal to acquire an osteoblast-like phenotype (104, 105).

Murine knockout models have shown that Wnt signaling may help to drive the osteogenic-like and chondrogenic-like differentiation of VSMCs. Conditional deletion of Msx1 and Msx2 reduces calcification of VSMCs in atherosclerotic murine models via reducing Wnt7b, Wnt5a, and Wnt2 signaling (106). And VSMC-specific deletion of the Wnt receptor, LRP6, protects against atherosclerotic calcification of VSMCs (107).

VSMCs can also differentiate into a chondrocyte-like state. Mice with loss of MGP develop extensive calcification in the large vessels and die a few months after birth due to rupture. These mice exhibit osteochondrogenic precursors that have the ability to differentiate into osteoblast-like and chondrocyte-like cells (108). Osteochondrogenic precursors exhibit decreased expression of SMC-specific genes such as SM22α and myocardin, and increased expression of Runx2. Osteogenesis is driven by increased activity of osterix, Msx2, and Wnt/b-catenin, while chondrogenesis is driven by decreased activity of Msx2 and increased activity of Sox9, a master regulator of chondrogenesis (109). Importantly, VSMCs have been shown to express both Runx2 and Sox9 *in vitro* and *in vivo*. While the expression of Runx2 appears to correlate with the onset of calcification *in vivo*, Sox9 expression is more widespread, which suggests Sox9 may regulateexpression of several ECM genes shared by both VSMCs and cartilage (110).

## A Common Denominator in the Cell Fate Switch

The phenotypic switch of a healthy vascular cell into a calcifying one requires the upregulation of genes and proteins that participate and regulate the calcification process. A common denominator in all forms of ectopic calcification is the enzyme tissue non-specific alkaline phosphatase (TNAP, in reference to the protein; *ALPL* is the gene encoding TNAP; NM_000478), which is both necessary and sufficient for the mineralization in physiological and pathological calcification (111, 112). *ALPL* has also been shown to be one of the earliest calcification-related genes upregulated during ectopic calcification *in vivo* (13). TNAP breaks down pyrophosphate (PPi), an endogenous inhibitor of calcification, to Pi, a building block necessary for mineralization; the extracellular PPi/Pi ratio drives the ectopic calcification process and TNAP is the key enzyme that regulates this balance (113). A sophisticated murine model was developed which specifically overexpresses *ALPL* in an X-linked manner in VSMCs using the *TAGLN* promoter, enabling a dose-effect of TNAP to be studied (114). In this model, medial-layer calcification occurred in a dose-dependent manner and was independent of alteration in serum levels of calcium, phosphate, or renal function in the mice, highlighting that TNAP activity alone can induce calcification. Similarly, this same group used a *Tie2* system to overexpress *ALPL* in endothelial cells and found a similar, though less severe, vascular calcification phenotype (115). Together, these *in vivo* models solidify the idea that TNAP activity alone is sufficient to promote ectopic calcification. Less well-defined are the various factors that induce the transcription of *ALPL* in the cells of the vasculature. Indeed, a large question remaining in the switch of a healthy to a calcifying cell is whether these shifts occur at the transcriptional level, or whether more large-scale epigenetic changes occur.

**Figure 1:**
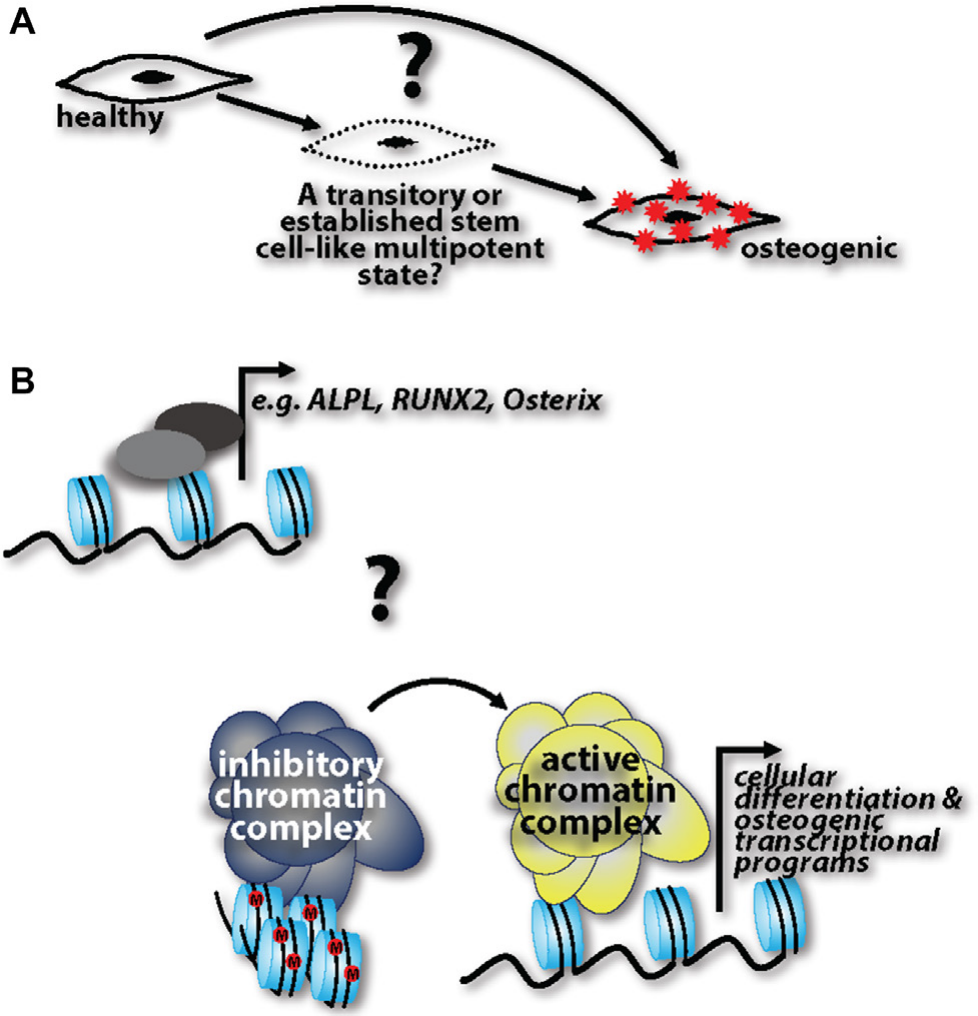
**(A)** Three possibilities are operative in ectopic calcification pathogenesis: healthy cells can directly acquire an osteogenic phenotype; there is a step-wise progression where cells transition through a progenitor-like state before acquiring an osteogenic phenotype; healthy cells de-differentiate into progenitor-like cells and are able to stay in this pluripotent state for some time, followed by a cue inducing them re-differentiate back to their initial state or transdifferentiate into calcifying cells. **(B)** Coinciding with these three possibilities, it is unknown whether these phenotypic transitions are regulated at the transcriptional level of osteogenic genes or whether there are more global epigenetic changes that alter the cell at a more global level

Other key questions remain (Figure 1): Do fully differentiated cells de-differentiate into progenitor-like cells and then readily acquire an osteogenic phenotype, or are de-differentiated cells able to stay in this pluripotent state for some time, and then depending on the cue, re-differentiate back to their initial state or transdifferentiate into calcifying cells? If the latter is the case, what are the cues that influence re-differentiation or transdifferentiation, respectively? This “MSC-like” window could be specifically targeted to halt disease progression. Cell lineage tracking genetic models specific to calcification processes are lacking. Harnessing these technologies would help to capture cells in the de-differentiated state and track their progression into a fully calcifying cell. And lastly, while evidence cited herein shows the ability of healthy cells to transition into calcifying cells, it is unclear whether these transitions happen at the transcriptional level of specific genes, such as *Runx2*, and *ALPL*, or whether there are more global epigenetic changes that are at play in this pathogenesis. Future studies addressing these questions with help identify druggable targets to harness, halt, or possibly even reverse ectopic calcification in the vasculature.

## Author Contributions

CS recommended the review topic, developed the direction and an outline of the review, provided content as well as extensive editing. LH and SS are postdoctoral fellows in CS’s group and performed extensive literature review and drafted the article. CS created the figure.

## Conflict of Interest Statement

The authors declare that the research was conducted in the absence of any commercial or financial relationships that could be construed as a potential conflict of interest.
